# Modeling squamous cell carcinoma development and malignant progression in mice

**DOI:** 10.1186/1753-6561-7-S2-K3

**Published:** 2013-04-04

**Authors:** Carlos Caulin

**Affiliations:** 1 Department of Head and Neck Surgery, The University of Texas M. D. Anderson Cancer Center, Houston, Texas, USA

## 

Squamous cell carcinoma (SCCs) of the skin and head and neck arise through the accumulation of genetic and epigenetic alterations that contribute to different stages of tumor progression and the metastatic potential of these tumors. Advanced SCCs are refractory to current therapies and exhibit high risk for relapse, and despite significant advances in prevention and in understanding the biology of SCCs, there are currently no effective treatments for patients with metastatic SCCs. Understanding the role of the molecular alterations found in SCCs may facilitate the design of novel therapies to treat these patients. The most frequently mutated gene in SCC of the skin and head and neck is the p53 gene, which is found mutated in up to 50-70% of the SCCs analyzed. The majority of the p53 mutations found in SCCs are missense mutations that result in the expression of altered forms of p53, some of which may promote tumorigenesis, indicating that p53 is not only a major tumor suppressor gene, but it may also be a primary oncogene in SCC. Genetically engineered mouse models are powerful tools to determine the role of genetic alterations found in human cancers. We generated a mouse model for sporadic head and neck cancer based on the focal activation of endogenous K-ras and p53 mutations in the oral epithelium. Using this model we demonstrated that the p53 gain-of-function mutation p53^R172H^, but not deletion of p53, contributes to oral tumor initiation, accelerates tumor growth and promotes malignant progression. Molecular analysis of tumors and cell lines derived from them determined that mutant p53 promotes the expression of mitotic genes and induces accelerated entry in mitosis, a mechanism that depends on oncogenic K-ras. These findings provide in vivo evidence for the oncogenic potential of mutant p53^R172H^ during SCC development and support clinical observations indicating that certain p53 mutations are associated with poor prognosis in SCC of the head and neck. I will discuss the potential implications of these findings in tumor progression, resistance to therapy and genomic instability associated with p53 mutations. As the oncogenic function of mutant p53 in this mouse model was dependent on K-ras mutations, we speculate that p53 mutants are activated in response to oncogenes. To address this question, I will further discuss the consequences of activating p53 gain- and loss-of-function mutations in the absence of additional genetic alterations in mouse models in which only p53 mutations are induced in stratified epithelia of the skin and the oral mucosa. In addition, novel mouse models generated in our laboratory are shedding new light into the function of genes that cooperate with p53 mutations in the early stages of SCC formation and during malignant progression and metastasis.

## Competing interests

There are no competing interests in this presentation.

